# Cushion plants as critical pioneers and engineers in alpine ecosystems across the Tibetan Plateau

**DOI:** 10.1002/ece3.7950

**Published:** 2021-07-27

**Authors:** Yi Wang, Jian Sun, Biying Liu, Jinniu Wang, Tao Zeng

**Affiliations:** ^1^ School of Life Sciences and School of Ecology State Key Lab of Biological Control Sun Yat‐sen University Guangzhou China; ^2^ State Key Laboratory of Tibetan Plateau Earth System Science (LATPES) Institute of Tibetan Plateau Research Chinese Academy of Sciences Beijing China; ^3^ College of Earth Sciences Chengdu University of Technology Chengdu China; ^4^ Synthesis Research Centre of Chinese Ecosystem Research Network Key Laboratory of Ecosystem Network Observation and Modelling Institute of Geographic Sciences and Natural Resources Research, Chinese Academy of Sciences Beijing China; ^5^ Chengdu Institute of Biology Chinese Academy of Sciences Chengdu China

**Keywords:** *Androsace* L., *Arenaria* L., cushion plant, ecosystem succession, Tibetan Plateau

## Abstract

Cushion plants are widely representative species in the alpine ecosystem due to their vital roles in influencing abiotic and biotic environments, ecological succession processes, and ecosystem engineering. Importantly, cushion plants, such as *Androsace* L. and *Arenaria* L., are considered to be critical pioneers of ecosystem health, restoration, and sustainability across the Tibetan Plateau. This is because cushion plants (a) show tenacious vitality and can modify regional climates, substrates, and soil nutrients in extreme environments; (b) facilitate relationships with the surroundings and maintain the diversity of aboveground and belowground communities; and (c) are highly sensitive to environmental changes and thus can indicate grassland ecosystem health and resilience in the context of global change.

## Graphical abstract

Cushion plants, such as *Androsace* L., have a median uplifting structure that creates “microspace” and a developed root system. The death of cushion plants provides sufficient fertilizer for surrounding plants.

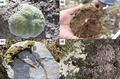

## 1 INTRODUCTION

**FIGURE 1:**
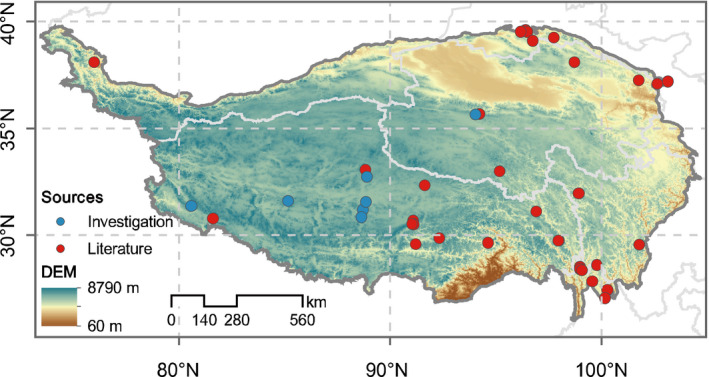
Research sites of cushion plants in the Tibetan Plateau collected from the literature and field investigation (Table S1)

Cushion plants, a key form of flora, comprise about 338 species within 34 plant families and are widely distributed in polar and alpine regions such as the South American Andes, Rockies, Tibetan Plateau, Alps, Tasmania, New Zealand, and Tierra del Fuego (Arredondo‐Núez et al., 2009; Meng et al., 2013). The Tibetan Plateau, which hosts 85 species of cushion plants, is a diversity hotspot of cushion plants that has received considerable attention from ecologists (Chen et al., 2017; Li et al., 1987). However, cushion plants in alpine ecosystems are at high risk because of global climate change (Alatalo et al., 2017; Chen et al., 2011). It has been documented that cushion plants are extremely vulnerable to global warming because of their fickle habitats (Michalet et al., 2014; Zhao et al., 2019). Furthermore, cushion plants are considered to be foundation species for vegetation succession because of their roles in modifying microenvironments in alpine ecosystems (Cavieres et al., 2016; Reid & Lortie, 2012). Therefore, it is essential to update our understandings of the responses of cushion plants to climate change (Figure 1).

## 2 THE CUSHION PLANT AS A KEY BUILDER OF ABIOTIC ENVIRONMENTS

**FIGURE 2:**
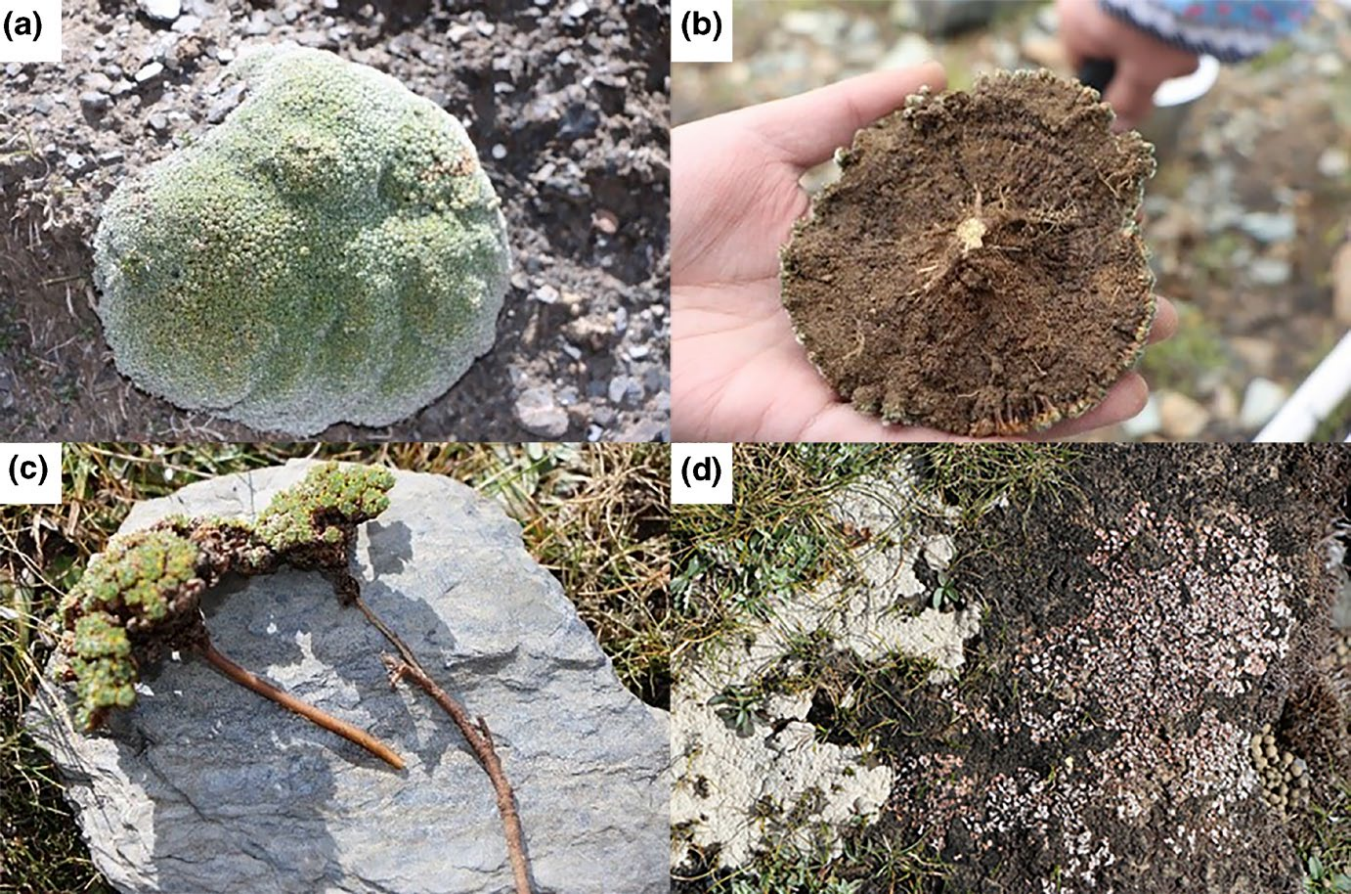
(a–c) Cushion plants, such as *Androsace* L., have a median uplifting structure that creates "microspace" and a developed root system. (d) The death of cushion plants provides sufficient fertilizer for surrounding plants

Cushion plants, for example, *Androsace* L. and *Arenaria* L., inhabit regions with altitudes higher than 4,000 m in the Tibetan Plateau and play a vital role in the development of modern flora and vegetation (Luo et al., 2018). The "microspace" formed by their convex structure (height: ~3–5 cm; diameter: ~20–30 cm) and the developed root system can function in heat, nutrient, and water preservation (Figure 2a–c) (Cavieres et al., 2006; Zhao et al., 2020). Cushion plants, which can persist for hundreds of years due to their tenacious vitality, function as ecological engineers by accelerating the enrichment of substrate nutrients (Figure 2d) (Yang et al., 2010). For instance, cushion plants can alleviate stress conditions and increase soil moisture, thereby increasing soil organic matter to levels higher than those that exist in the open microhabitat (Cavieres et al., 2006, 2007). Moreover, the special structure and strong adaptability of cushion plants prevent damage by wind and water erosion and maintain the warmth of extreme regions (Byers et al., 2006). Ultimately, dead cushion plants provide sufficient substrate into the soil and facilitate nutrient cycling.

## 3 CUSHION PLANTS AS CRITICAL FACILITATORS FOR BIOTIC ENVIRONMENTS

**FIGURE 3:**
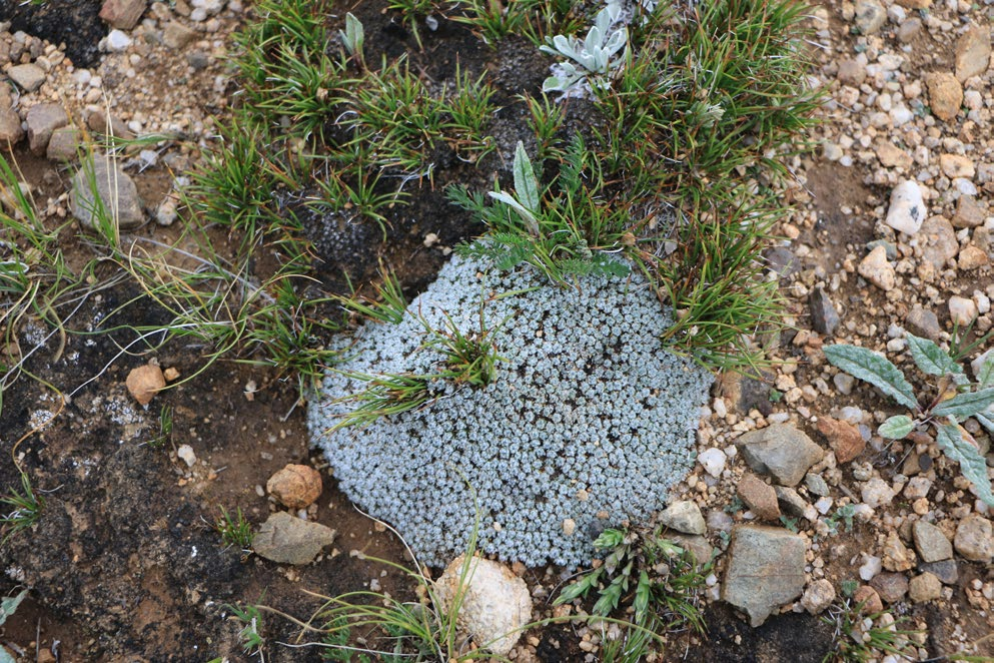
Cushion plants promote the propagation of other plants, such as *Leontopodium*, *Carex*, and *Artemisia* species

Cushion plants provide physical protection in extreme environments and promote the growth and reproduction of surrounding species, especially pioneer species (Cavieres et al., 2006; Francisco et al., 2020). Thus, cushion plants exhibit interspecific facilitation, which may be beneficial to maintaining species diversity (Erfanzadeh et al., 2020). For example, it has been reported that cushion plants can provide an environment favorable for supporting annual herbs (Liu et al., 2016; Schöb et al., 2016) (Figure 3). In addition, cushion plants can influence belowground processes by enhancing the activity of soil microorganisms and enhancing fungal communities to increase the absorption area of roots and maintain microbial community diversity (Casanova‐Katny et al., 2011; Chang et al., 2018; Wang et al., 2020). Because microbial communities are assembled and affected by cushion plants, microbes can share the nutrient pools in the rhizosphere with cushion plants (Chang et al., 2018).

## 4 CUSHION PLANTS AS INDICATORS OF ECOSYSTEM HEALTH AND RESILIENCE

**FIGURE 4:**
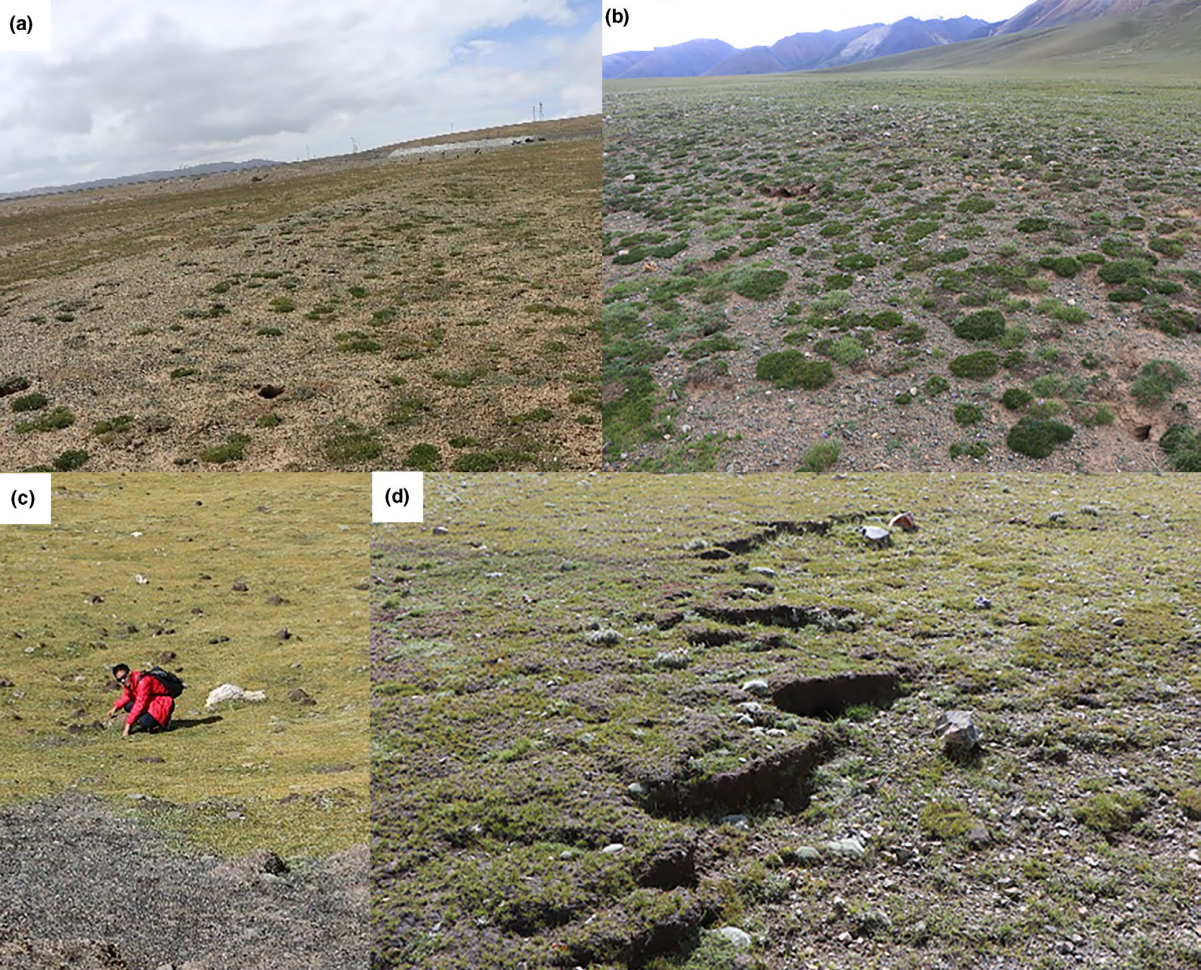
(a, b) *Arenaria* L. inhabit degraded grasslands. (c, d) *Androsace* L. inhabit the alpine grassland ecosystem at an altitude of ~4,700 m

Cushion plants are extremely sensitive to global change (e.g., climate, nitrogen deposition, species invasion, and land use) and thus may indicate ecosystem health and resilience (Gorsuch et al., 2001). Specifically, in the context of alpine grassland ecosystem degradation, cushion plants play an important role in improving abiotic and biotic environments and maintaining the stability of the alpine ecosystem (Erfanzadeh et al., 2020; Luo et al., 2018) (Figure 4). Furthermore, cushion plants act as a safe island for settled plants, which can reduce species competition and improve adaptability among species (Meng et al., 2013). The promoting effects of cushion plants could increase with environmental stress. For instance, cushion plants at low altitudes in the Andes show strong promotion effects (which could be due to the influence of strong water limitation) and reflect high resilience on the community scale (Cavieres et al., 2006).

## 5 CONCLUSIONS

Cushion plants are key species for maintaining the stability of the grassland ecosystem across the Tibetan Plateau because of their vital roles in modifying the surrounding environment and nutrient cycling processes. Cushion plants promote species reproduction of surrounding plants and are hotspots for plant and microbial diversity. Notably, cushion plants are essential indicators of ecosystem health and sustainability and play important roles in the restoration of alpine grassland ecosystems under the influences of global change. Therefore, cushion plants are both important pioneers and ecosystem engineers in the alpine ecosystems of the Tibetan Plateau.

## CONFLICT OF INTEREST

The authors declare no conflict of interest.

## AUTHOR CONTRIBUTIONS

**Yi Wang:** Conceptualization (lead); Data curation (equal); Investigation (equal); Visualization (lead); Writing‐original draft (lead); Writing‐review & editing (lead). **Jian Sun:** Conceptualization (equal); Data curation (lead); Investigation (lead); Writing‐review & editing (equal). **Biying Liu:** Investigation (equal); Writing‐original draft (supporting); Writing‐review & editing (supporting). **Jinniu Wang:** Writing‐original draft (supporting); Writing‐review & editing (supporting). **Tao Zeng:** Conceptualization (equal); Writing‐original draft (supporting); Writing‐review & editing (supporting).

## Supporting information

Appendix S1

## Data Availability

Table S1 associated with this article is deposited on the Dryad Digital Repository (https://doi.org/10.5061/dryad.4b8gthtcz).
