# Supplementary material for: Cushion plants as critical pioneers and engineers in alpine ecosystems across the Tibetan Plateau
Source: Ecol Evol. 2021 Jul 27;11(17):11554–8. doi: 10.1002/ece3.7950 (PMC8427563; doi:10.1002/ece3.7950)
Supplement: Supplementary file 1 — Appendix S1 [file ECE3-11-11554-s001.docx]

# Table S1

| Lon | Lat | Altitude | Cushion plants | Sources | Reference |
| --- | --- | --- | --- | --- | --- |
| 99.00012 | 28.4521 | 4427 | *Arenaria polytrichoides* | Literature | Chen et al. (2020) |
| 99.00105 | 28.47829 | 4732 | *Arenaria polytrichoides* | Literature | Chen et al. (2020) |
| 76 | 38.08333 | 4750 | *Arenaria polytrichoides; Arenaria bryophylla; Arenaria pulvinata* | Literature | Li et al. (2021) |
| 98.99 | 28.48 | 4640 | *Arenaria polytrichoides* | Literature | Zhang et al. (2020) |
| 98.00639 | 29.71972 | 5125 | *Arenaria densissima* | Literature | Xu et al. (2020) |
| 99.58694 | 27.7925 | 4380 | *Arenaria oreophila* | Literature | Xu et al. (2020) |
| 9.942778 | 28.18361 | 4614 | *Arenaria polytrichoides* | Literature | Xu et al. (2020) |
| 100.2989 | 27.41444 | 4229 | *Arenaria smithiana* | Literature | Xu et al. (2020) |
| 99.80667 | 28.57139 | 4365 | *Thylacospermum ceaspitosum* | Literature | Xu et al. (2020) |
| 96.433 | 39.59 | 3696 | *Thylacospermum ceaspitosum* | Literature | Wang et al. (2020) |
| 86.821 | 43.117 | 3772 | *Thylacospermum ceaspitosum* | Literature | Wang et al. (2020) |
| 94.25 | 35.65 | 6178.6 | *Thylacospermum caespitosum; Androsace tangulashanensis* | Literature | Zhao et al. (2020) |
| 96.433 | 39.59 | 3696.5 | *Thylacospermum ceaspitosum* | Literature | Wang (2014) |
| 103.169 | 37.181 | 3689 | *Thylacospermum ceaspitosum* | Literature | Wang (2014) |
| 86.821 | 43.117 | 3772.6 | *Thylacospermum ceaspitosum* | Literature | Wang (2014) |
| 103.169 | 37.181 | 3689 | *Arenaria kansuensis* | Literature | Wang (2014) |
| 97.75567 | 39.237 | NA | *Thylacospermum ceaspitosum* | Literature | Zhao et al. (2018) |
| 97.498 | 43.78 | NA | *Thylacospermum caespitosum* | Literature | Zhao et al. (2018) |
| 98.98778 | 28.37083 | NA | *Arenaria polytrichoides; Chionocharis hookeri* | Literature | Chang et al. (2018) |
| 102.6667 | 37.06667 | 3800 | *Thylacospermum caespitosum* | Literature | Jiang (2014) |
| 96.5 | 39.5 | 4260 | *Thylacospermum caespitosum* | Literature | Jiang (2014) |
| 96.41667 | 39.58333 | 3696 | *Thylacospermum caespitosum* | Literature | Jiang (2014) |
| 86.81667 | 43.11667 | 3772 | *Thylacospermum caespitosum* | Literature | Jiang (2014) |
| 91.64497 | 32.30857 | NA | *Arenaria polytrichoides; Androsace tapete; Arenaria serpyllifolia* | Literature | Qin & Xie (1980) |
| 81.62758 | 30.75244 | NA | *Arenaria polytrichoides; Androsace tapete; Arenaria serpyllifolia* | Literature | Pan et al. (1977) |
| 96.43611 | 39.58889 | 3733 | *Thylacospermum caespitosum* | Literature | Cui (2016) |
| 96.43306 | 39.59 | 3696.7 | *Thylacospermum caespitosum* | Literature | Cui (2016) |
| 96.505 | 39.50306 | 4260 | *Thylacospermum caespitosum* | Literature | Cui (2016) |
| 86.82194 | 43.11694 | 3772.5 | *Androsace tapete; Thylacospermum caespitosum* | Literature | Cui (2016) |
| 99.01111 | 28.39 | 4543.8 | *Arenaria polytrichoides* | Literature | Cui (2016) |
| 98.74528 | 38.08722 | 4145.6 | *Arenaria kansuensis* | Literature | Cui (2016) |
| 101.7831 | 37.21972 | 3767.9 | *Arenaria kansuensis* | Literature | Cui (2016) |
| 91.22 | 29.55 | 4700 | *Androsace tapete* | Literature | Cui (2016) |
| 92.345 | 29.82806 | 5100 | *Androsace tapete* | Literature | Cui (2016) |
| 91.10389 | 30.64806 | 5102 | *Androsace tapete* | Literature | Cui (2016) |
| 94.65389 | 29.61306 | 5000 | *Androsace tapete; Arenaria densissima* | Literature | Cui (2016) |
| 96.77208 | 39.08631 | 4137 | *Thylacospermum caespitosum* | Literature | Liu et al. (2016) |
| 88.85348 | 33.04397 | 5014 | *Androsace tapete* | Literature | Zhao et al. (2015) |
| 95.18311 | 32.96625 | 4800 | *Sibbaldia tetrandra* | Literature | Chen et al. (2015) |
| 98.93253 | 31.93647 | 5050 | *Arenaria polytrichoides* | Literature | Chen et al. (2015) |
| 98.9325 | 31.93653 | 5050 | *Chionocharis hookeri* | Literature | Chen et al. (2015) |
| 96.93694 | 31.08925 | 4400 | *Thylacospermum caespitosum* | Literature | Chen et al. (2015) |
| 97.98381 | 29.71203 | 5000 | *Arenaria lancangensis* | Literature | Chen et al. (2015) |
| 97.98383 | 29.71197 | 5000 | *Arenaria densissima* | Literature | Chen et al. (2015) |
| 101.8038 | 29.51803 | 4200 | *Potentilla articulata* | Literature | Chen et al. (2015) |
| 99.80619 | 28.57244 | 4500 | *Arenaria lancangensis* | Literature | Chen et al. (2015) |
| 99.08667 | 28.33125 | 4700 | *Potentilla articulata* | Literature | Chen et al. (2015) |
| 99.08667 | 28.33125 | 4700 | *Arenaria polytrichoides* | Literature | Chen et al. (2015) |
| 100.1808 | 27.03265 | 4200 | *Arenaria oreophila* | Literature | Chen et al. (2015) |
| 91.13333 | 30.48333 | 4950 | *Androsace tapete* | Literature | He et al. (2014) |
| 96.19356 | 39.50572 | 3762 | *Krascheninnikovia compacta; Thylacospermum caespitosum* | Literature | Liu (2014) |
| 91.05 | 30.5 | 4800 | *Androsace tapete* | Literature | Li et al. (2013) |
| 88.91686 | 32.71939 | 4954 | *Androsace tapete; Arenaria serpyllifolia* | Investigation | Transect |
| 88.71447 | 31.16291 | 4785 | *Androsace tapete; Arenaria serpyllifolia* | Investigation | Transect |
| 88.87331 | 31.52624 | 4554 | *Androsace tapete; Arenaria serpyllifolia* | Investigation | Transect |
| 80.60144 | 31.35078 | 4800 | *Androsace tapete; Arenaria serpyllifolia* | Investigation | Transect |
| 85.18301 | 31.57306 | 5233 | *Androsace tapete; Arenaria serpyllifolia* | Investigation | Transect |
| 88.67034 | 30.82373 | 4750 | *Androsace tapete; Arenaria serpyllifolia* | Investigation | Transect |
| 94.06518 | 35.6235 | 4751 | *Androsace tapete; Arenaria serpyllifolia* | Investigation | Transect |

# References

Chang, S., Chen, J., Su, J., Yang, Y., & Sun, H. (2018). Seasonal comparison of bacterial communities in rhizosphere of alpine cushion plants in the Himalayan Hengduan Mountains. *Plant Diversity*, 40(5), 209-216.

Chen, J., Schöb, C., Zhou, Z., Gong, Q., Li, X., Yang, Y., Li, Z., & Sun, H. (2015). Cushion plants can have a positive effect on diversity at high elevations in the Himalayan Hengduan Mountains. *Journal of Vegetation Science*, 26(4), 768-777.

Chen, J., Zhang, Y., Zhang, H., Schb, C., & Hang, S. (2020). The positive effects of the alpine cushion plant Arenaria polytrichoides on insect dynamics are determined by both physical and biotic factors. *Science of The Total Environment*, 762(68), 143091.

Cui, H. M. (2016). Morphological and structural features of six typical alpine cushion plants ,leaves adapt to the alpine environment

He, Y., Xu, X., Kueffer, C., Zhang, X., & Shi, P. (2014). Leaf litter of a dominant cushion plant shifts nitrogen mineralization to immobilization at high but not low temperature in an alpine meadow. *Plant & Soil*, 383(1-2), 415-426.

Jiang, X. (2014). Phenotypic variation of Alpine cushion plants and its effects on species interaction and community composition

Li, C. S., Ma, Q. W., & Yang, J. (2021). Cushion plants in the West Kunlun Mountains. *Life Word*, 130-37.

Li, R., Luo, T., Tang, Y., Du, M., & Zhang, X. (2013). The altitudinal distribution center of a widespread cushion species is related to an optimum combination of temperature and precipitation in the central Tibetan Plateau. *Journal of Arid Environments*, 8870-77.

Liu, X. (2014). Studies on the ecosystem engineering effect of cushion plants in alpine cold desert at the northern margin of Tibetan Plateau.

Liu, X., Sun, X., & Tian, Q. (2016). Effect of cushion plant Thylacospermum caespitosum Camb. on species diversity of community. *Acta Ecologica Sinica*, 36(10), 2905-2913.

Pan, J. T., Chang, H. Z., & Liu, S. W. (1977). The alpine plants in Mapamyum co district and on the south slope of Pengzhe feng of Gengdise shan. *Journal of Integrative Plant Biology*, 19(2), 138-146.

Qin, Z. Y., & Xie, W. Z. (1980). Observations on the morphology and ecology of the cushion plants in Togme region of northern Xizang. *Journal of Integrative Plant Biology*, 22(2), 177-181.

Wang, C., Michalet, R., Liu, Z., Jiang, X., Wang, X., Zhang, G., An, L., Chen, S., & Xiao, S. (2020). Disentangling large- and small-scale abiotic and biotic factors shaping soil microbial communities in an alpine cushion plant system. *Frontiers in Microbiology*, 11925.

Wang, C. Y. (2014). Studies on the ecosystem engineering effect of cushion plants in alpine cold desert at the northern margin of Tibetan Plateau

Xu, B., Sun, W., & Li, Z. (2020). Karyological Study of Five Cushion Plants of Caryophyllaceae in Qinghai-Tibet Plateau. *Acta Botanica Boreali-Occidentalia Sinica*, 40(7), 1157-1163.

Zhang, Y., Wang, S. W., He, X., Yang, Y., & Sun, H. (2020). Altitudinal variation in flowering area and position and their ecological significances of an alpine cushion Arenaria polytrichoides, a gynodioecious herb. *Chinese Journal of Plant Ecology*, 44(11), 1154-1163.

Zhao, H., Guo, K., Yang, Y., & Qiao, X. (2015). Age Determination and Growing Patterns of the Cushion Plant Androsace tapete in the Tibetan Plateau. *Mountain Research*, 4(473-479),

Zhao, R., Zhang, H., & An, L. (2020). Spatial patterns and interspecific relationships of two dominant cushion plants at three elevations on the Kunlun Mountain, China. *Environmental Science and Pollution Research*, 27(17), 17339-17349.

Zhao, R. M., Zhang, H., & An, L. Z. (2018). Thylacospermum caespitosum population structure and cushion species community diversity along an altitudinal gradient. *Environmental Science and Pollution Research*, 25(29), 28998-29005.
